# Editorial: Harnessing digital health innovations to improve healthcare delivery in Africa: Progress, challenges and future directions

**DOI:** 10.3389/fdgth.2023.1037113

**Published:** 2023-03-24

**Authors:** Olushayo Oluseun Olu, Humphrey Cyprian Karamagi, Joseph Chukwudi Okeibunor

**Affiliations:** ^1^ Office of the Representative, World Health Organization Country Office, Juba, South Sudan; ^2^ Assistant Regional Director Cluster, World Health Organization Regional Office for Africa, Brazzaville, Republic of Congo

**Keywords:** digital health innovations, digital health, eHealth, health systems, healthcare delivery, universal health coverage, Africa

**Editorial on the Research Topic** Harnessing digital health innovations to improve healthcare delivery in Africa: Progress, challenges and future directions

## Introduction

Diseases, environmental, political and security shocks coupled with challenges in governance and financing of health services continue to weaken the capacity of African countries to deliver good health services and attain global health and development goals such as Universal Health Coverage (UHC), the Sustainable Development Goals (SDGs), the International Health Regulations 2005 (IHR 2005) and the Global Health Security Agenda. The weak capacity is attributable to both intrinsic and extrinsic challenges which continue to decimate the ability of the health system of these countries to perform optimally. The intrinsic challenges are linked to the quality and quantities of investments in the health system building blocks such as health governance, financing, human resources for health, health information management and essential medicines and medical supplies which hamper the ability to deliver equitable and quality essential health services that people need (1, 2). Coupled with this are extrinsic challenges including social, environmental, cultural, economic, political and physical barriers which limit access to health care (3, 4). The ongoing COVID-19 pandemic, recurring outbreaks of Ebola Virus Disease (EVD), Cholera, Measles and now Monkeypox coupled with perennial incidents of conflicts and natural disasters have further aggravated these health system challenges (5–7).

Attainment of global health and related development goals on the continent thus require innovative ways of applying existing investments and new innovations to overcome the foregoing challenges and deliver good quality essential health services (8)—a role that Digital Health Innovations (DHIs) can effectively play (9). Globally and in Africa, DHIs have been successfully deployed to address medical challenges in both clinical and public health medicine at the individual, community and health facility levels (10, 11). They have been shown to improve efficiency in health service delivery, reduce the cost of healthcare, improve access to health information and provide timely health information for evidence-based decision making (12). However, the implementation of DHI programmes has been constrained by several factors on the continent. While many African countries have developed and adopted digital health/eHealth technology strategies, their effective implementation is constrained by inadequate digital health governance and legal frameworks, and weak institutional capacity for planning, implementation, supervision, monitoring, evaluation and sustainable funding of such strategies. Furthermore, mushrooming of multiple and parallel digital health/eHealth initiatives most of which are not interoperable, the dearth of digitally educated health workers and lack of data ownership and security frameworks continue to constrain the effective roll-out and sustainability of DHIs. Other challenges include unreliable information and electronic communication and sustainable power infrastructures particularly in the rural and hard-to-reach areas of the continent (13).

Successful deployment of DHIs in Africa would require better understanding of the factors which facilitate or hinder effective and sustainable implementation of such innovations from a practical and evidence-based point of view. The aim of this Research Topic is therefore to explore the progress, challenges and future of how DHIs have been successfully deployed to overcome the foregoing health system challenges on the continent. The Topic focuses on identifying innovative concepts that address existing challenges, sharing practical field experiences on what has worked well, what did not work so well and how these innovations can be adapted to the context of developing countries sustainably and cost-effectively. This editorial summarizes the articles published in the Research Topic and draw conclusions which could improve effective roll out of DHIs in Africa moving forward.

## Key highlights of published articles

Eight articles including this editorial, are published in this Research Topic. This comprise four original researches, two reviews and one study protocol addressing applications of DHIs to broad areas of health system strengthening, health information management, community-based health service delivery, interoperability of various platforms and institutional capacity building.

The Research Topic begins with an original research article on a neonatal outcome audit using digital tools in a low-resource neonatal unit in Malawi by Mgusha et al. The study collected patient admission and outcome data digitally using an application called NeoTree which was collaboratively developed by the Ministry of Health and end users based on existing data collection systems such as the District Health Information Management System. The findings conveyed that the mobile application facilitated collection of more complete, high-quality and reliable admission and outcome information resulting in improvements in quality of care in the areas of perinatal management of HIV and syphilis and management of babies born with hypothermia in the unit. Furthermore, the dashboard which was established using the mobile application was used to conduct real-time neonatal audits providing opportunities for timely corrective actions. The study recommended scale up of this digital data collection system and measurement of its impact on neonatal health outcomes in future.

Ibeneme et al. in their article reviewed a Digital Health Platform (DHP) which is being supported by the African Regional Office of the World Health Organization (WHO/AFRO). The platform is an open-source, comprehensive, modular and flexible digital solution for electronic management of health events at the individual patient level. Its main advantages include the ability to enforce use of care standards, enable continuous beneficiary use, eliminate interoperability challenges of multiple data collection systems, use existing hardware for start-up and address the often-vertical external partner facilitated and programme specific Electronic Health Record Systems on the continent. It is thus a tool not just for data harvesting, but more importantly also supports improved access to, and quality of services provided. Challenges of this platform include inadequate in-country capacity, erratic power supply, and managing the change process for both users, beneficiaries and other partners supporting similar systems. The paper recommended strong partnerships, capacity building, deployment of sustainable roll-out models and establishment of monitoring and evaluation frameworks as means to fast-track full and effective roll out of the platform.

Fredriksson et al. published a study which used digital methods to predict maternal health services utilization pattern in a maternal health programme called “*Uzazi Salama”* in Zanzibar, Tanzania. In this study, Community Health Workers (CHWs) used a smartphone application to collect maternal health data which was then used to develop a model to predict the delivery location of women who were enrolled into the programme. The findings showed that the data/model facilitated accurate prediction of the delivery location of a significant majority of the women that were already enrolled into the programme and could also be used to predict the delivery location of new entrants. Such information is useful for cost-effective planning of maternal health services programmes in low-resource settings. The study concluded that while these models were developed from data collected from an existing project, the framework could be adapted to suit data collected from new projects within and outside maternal health.

In their paper, Kipruto et al. presented the protocol of a study which aims to document the use of DHIs to strengthen health systems in sub-Saharan Africa in the last ten years. The proposed study is aimed at identifying, categorizing, describing and mapping DHIs to relevant building blocks of the health systems. It also seeks to describe the challenges and gaps in the implementation of DHIs on the continent with a view to providing evidence to stakeholders on cost-effective allocation of DHI resources.

Owoyemi et al. reviewed the barriers encountered and methods that worked in the implementation of mHealth interventions at community and primary health care settings in Africa. They identified among others, limited wireless data availability, erratic electricity supply, limited skills of community health workers in the use of DHI tools and instability of mHealth products resulting in software crashes or freezes as the critical challenges to the implementation of DHI programmes at the community level. The authors described innovative ways that worked well to address these challenges such as the use of solar energy, training and equipping of health sector information management technicians to support trouble shooting at the community level and use of offline mHealth solutions among others. The involvement of stakeholders in the design and implementation of mHealth solutions was another innovation. The review recommended further research to document the impact of mHealth solutions on health outcomes as well as more investments in development of infrastructure which could facilitate increased uptake and effectiveness of DHIs in Africa.

In their research paper, Bello et al. examined the reliability of the GPS coordinates of supervisors who conduct health facility-based supportive supervision in a World Health Organization (WHO) Immunization programme in Nigeria. The study compared and interrogated the level of deviation between the actual position of the mobile phones (and supervisors) and their recorded GPS coordinates with a view to identify the most reliable brands of mobile devices for programme use. It showed a 1.6% deviation which was largely associated with the quality of the phones, the training and skills provided to the phone users. The authors concluded that while the use of mobile phones for supervision facilitated availability of real-time data, timely and informed decision making, accountability and transparency of the supervision process, it was constrained by the poor quality of the mobile phones used and the skills of the phone users. They recommended procurement of higher quality mobile devices for programme use and improved training of the phone users.

Millimouno et al. evaluated a blended training programme which combined e-learning with the traditional face-to-face type of learning for health workers in Guinea in 2021. The study assessed the completion rate, the reason for drop-out and the impact of the course on the skills of the course participants. The findings showed a fair course completion rate (67%–69%) in general and identified lack of technological skills, breakdown or loss of information and communication equipment, lack of access to good internet connection and travel to areas where internet connectivity is unstable as the key factors which determined drop-out from these blended learning programmes. The study concluded that blended learning programmes had fair success rates and had positive impact on changing participants work behaviours and should thus be encouraged.

## Conclusion

As demonstrated by the evidence presented in this Research Topic, there is no doubt that DHIs have great potential to strengthen health systems, improve the quality, quantity and accessibility to healthcare services towards attaining key global health and related development goals in Africa despite the associated challenges. The practical field experiences which have been gleaned from the articles in this research topic have demonstrated that although daunting, the challenges which impede sustainable and effective roll-out of DHIs are surmountable. The findings and recommendations of the articles are critical lessons which could shape effective and sustainable roll out of DHIs in the African context and on their basis, we propose a few recommendations.

First, new DHI programmes should be integrated into existing health system development processes at the planning stages to facilitate cost savings, improve scalability and enhance interoperability. Second, community-based DHIs should be easy to use, powered by appropriate local technology and stakeholders should be involved in its design and deployment. Third, more investments should be made to ensure sustainable and appropriate technology to facilitate wider access to stable internet connectivity, mobile telephone network and electricity supply particularly in the remote and rural areas of Africa. Fourth, more research to evaluate and document the impact of DHIs on public health outcomes should be conducted on the continent. Fifth, further studies which describe the application of DHIs to support the preparedness for, response to and recovery from ongoing pandemics and emerging epidemics such as COVID-19, EVD, cholera, measles, Monkeypox and the numerous natural and man-made disasters are required. Such studies should explore the possibility of using DHIs and the One Health approach to integrate human and animal health and ecosystem data to facilitate timely detection and response to outbreaks of zoonotic diseases and climate related public health emergencies. Finally, we call on all stakeholders involved with strengthening health systems and implementation of DHIs in Africa to entrench the above lessons and recommendations in the design and implementation of new DHI programmes and revision of the existing ones.
